# Impacts of Urban Agriculture on the Determinants of Health: Scoping Review Protocol

**DOI:** 10.2196/resprot.9427

**Published:** 2018-03-27

**Authors:** Pierre Paul Audate, Melissa A Fernandez, Geneviève Cloutier, Alexandre Lebel

**Affiliations:** ^1^ Graduate School of Land Management and Urban Planning Laval University Québec, QC Canada; ^2^ Institute of Nutrition and Functional Foods Laval University Québec, QC Canada; ^3^ School of Nutrition Laval University Québec, QC Canada; ^4^ Evaluation Platform on Obesity Prevention Quebec Heart and Lung Institute Québec, QC Canada

**Keywords:** urban agriculture, health, determinants of health, scoping review

## Abstract

**Background:**

Since the 1990s, urban agriculture (UA) has contributed to improving food security in low- and middle- income countries. Now, it is implemented as a multifunctional intervention that can influence various determinants of health (eg, food security, social relationships). Studies of interest stem from several research disciplines, use a wide range of methods, and show results that are sometimes inconsistent. Current studies have not summarized the overall effects of UA on health and its determinants.

**Objective:**

The objective of this protocol is to develop a research strategy for a scoping review that characterizes studies of beneficial and adverse impacts of UA on health and its determinants in a wide range of disciplines.

**Methods:**

Initially, with the help of a library specialist, a list of publications will be obtained through a systematic search of seven electronic bibliographic databases: PubMed, Embase, MEDLINE (Embase), CINAHL Plus with full text, Academic Search Premier (EBSCO host), CAB Abstract (Ovid), and Web of Science. Secondly, a three-step screening by two independent reviewers will lead to a list of relevant publications that meet eligibility and inclusion criteria. Finally, data on the bibliography, type of participants, type of study, results of study, and countries will be extracted from included articles and analyzed to be presented in a peer-reviewed article.

**Results:**

The findings are expected to identify research gaps that will inform needs for UA research in specific fields (eg, mental health), among certain population groups (eg, adults) or within different economic contexts (eg, low-, middle-, or high-income countries). Furthermore, the findings are expected to identify knowledge gaps and direct future research needs.

**Conclusions:**

This is an original study that seeks to integrate beneficial and adverse effects of UA on health at different level of influence (individuals, households, and community) in order to facilitate a better understanding of UA impacts. This protocol is a first of its kind and is expected to lead to a characterization of UA impacts based on sociodemographic profiles of participants and income levels of the studied countries. This will be relevant for policy makers and UA practitioners.

## Introduction

### Background

Since the 1990s, urban agriculture (UA) has been a strategy contributing to improving income and food security for individuals and households in low- and middle- income countries, particularly in Africa [1-5]. In cities such as Dar-es-Salaam, Tanzania and Bamako, Mali, UA provides more than 30% of the city’s vegetable needs and generates wages equivalent or higher than civil servants or unskilled construction workers [5]. In high-income countries, UA has contributed to food security in times of emergency or economic crisis [6-8]. For example, it is documented that countries in North America and Europe have encouraged their citizens to engage in UA activities during the first and second World Wars in response to pressures on the food supply [9,10]. Beyond its traditional purposes (food security and income improvement), UA is now considered as a multifunctional intervention [11,12]. It is part of health promotion strategies [13,14], urban planning [12], and/or global policies to develop sustainable city food systems [15,16]. It can also play an important role in the availability of green infrastructure and biodiversity in the urban environments [17]. Its function in the recycling of urban organic waste is also recognized [18]. In some contexts, it is perceived and practiced by urban dwellers to reduce the ecological footprint of the food industry [12]. It is supported by a range of actors including health professionals [19], government agencies, community groups, and researchers [20]. In general, it can be viewed as small areas used in cities for agricultural production or to raise animals for domestic consumption or local sales [21].

UA as an intervention can have social and economic impacts on individuals, households, and/or an entire community by directly influencing health or its determinants. It can influence food security, mental or physical health, or social relationships at different population levels. A significant number of studies have already attempted to demonstrate the contribution of UA to food security [22,23] by assuming an association between UA and access to food [24] or its association with improved household nutrition through consumption of fresh fruits and vegetables [25]. In addition, engagement in UA may improve physical activity and contribute to well-being and health by reducing stress [26,27]. However, the effects of UA on health and its determinants remain inconsistent. Many of these studies have been criticized for their lack of empirical evidence. For example, among studies that have shown UA contribution to food security at individual or household levels, some are often criticized because of poor data quality or lack of methodological rigor [28,29].

Other studies have focused on the negative effects of UA. For example, several studies highlight the potential public health risks associated with UA [19] by addressing concerns related to urban soil and water contamination. Some have raised concerns about the presence of heavy metals in UA soils or harvested crops [30-33] that may have implications for food safety. In fact, traces of heavy metals can be found in vegetables and fruit grown in urban areas, representing a health risk for individuals who consume such products [33,34]. On the other hand, the potential effects of UA products from contaminated soils on humans are unclear. The concentration of heavy metals in soil does not necessarily reflect heavy metal concentrations in harvested crops and the utilization of these crops does not inevitably represent a risk to human health [35,36]. Nevertheless, it is important to note that UA has potential public health risks, which need to be documented.

Although some systematic reviews have been conducted on UA and health, specifically food security and wellbeing [27], there are no reviews that refer to the adverse effects of UA. To our knowledge, most of these types of studies have not considered a holistic approach that includes beneficial and adverse impacts of UA. Three systemic reviews [28,37,38] have examined the contribution of UA to one type of determinant of health in a specific context; food outcomes in low- or middle-income countries. Two of them: Warren et al [28] and Poulsen et al [37] recommended new research due to poor quality and heterogeneity of the primary studies included. Although both studies have considered food security as an analytical framework, they only had four included studies in common. Poulsen et al [37] only included studies conducted in Africa, even though “region” was not part of the inclusion criteria. In contrast, Warren et al [28] included studies from other geographic locations. The differences may be due to a lack of consistency in research strategy or differences in their selection criteria. Korth et al [38] targeted studies in countries with similar characteristics, low- and middle-income countries, and failed to identify any studies. This reinforces our argument about a lack of consistency in UA contribution to food security in the systematic review processes. One of the common points between the three reviews was the absence of high-income countries in their analysis.

The consideration of high-income countries in literature reviews of interventions similar to UA is not new. Other systematic studies have already evaluated gardening or school gardening, which to some extent are similar interventions to UA. These studies do not allow to draw conclusions about the impacts of UA on health. For example, Ohly et al [39] used a mixed methods approach to measure the impacts of school gardens on health and well-being in high-income countries. However, the assessed studies were qualified as low or moderate quality based on the authors' criteria. While methodological weaknesses were also reported for the included quantitative studies, the qualitative studies were described as ideological aspirations. Nicklett et al [40] used the same concept of gardening to demonstrate its association with physical health in high-income countries. Yet, like Ohly et al [39], the review identified methodological weaknesses in the primary studies included, which limit conclusions on a possible impact of gardening activities on physical health.

At this time, current studies have not been able to draw definite conclusions on the effects of UA on specific determinants of health or health in general. Given that UA is a multidisciplinary topic (eg, nutrition, agriculture, urban planning), it may be better to address it first in a more general systematic process such a scoping review and consider a broader impact outcome like health prior to engaging future systematic reviews.

With this scoping review we seek to identify evidence from peer reviewed literature that demonstrates beneficial and adverse impacts of UA on the determinants of health according to countries’ income level as defined by the World Bank [41]. The determinants of health are defined as socioeconomic factors that influence health [42]. We aim to identify knowledge gaps and facilitate a better understanding of the global impact of UA on health and its determinants by considering the following two research questions:

1. What are the impacts of UA on health and its determinants?
2. How do these impacts differ according to countries’ income level or sociodemographic characteristics of studied participants?

Conceptually, by answering these questions, we will have a better understanding of how UA as an intervention can affect different health outcomes such as food security, nutrition, social relationships, physical or mental health. Furthermore, we are interested in categorizing these outcomes according to level of influence (individual, household, and community) and countries’ income level (high-, middle-, and low-income). The findings will allow us to draw a global picture of the potential impacts of UA on health present in the existing literature. Identifying research gaps will also allow researchers and policy makers to make informed decisions about future UA research needs and implications for public policy.

### Objective

The specific objectives of this study are:

1. To identify UA impacts on health and its main determinants
2. To characterize the results according to population and country income levels

## Methods

This scoping review will follow the five steps described by Arksey and O'Malley [43] for similar studies with improvements suggested by Levac et al [44]:

1. Identification of the research questions (listed above)
2. Identification of relevant studies
3. Selection of relevant and reliable studies
4. Data extraction from included studies
5. Collating, summarizing, and reporting the findings

### Identification of Relevant Studies:

This scoping review will use the method suggested by Aromataris and Riitano [45] to construct a strategy that can help us target relevant publications on UA impacts on health and its determinants. First, we will identify keywords that are related to our main research questions. To identify keywords, elements of a modified PICOS framework (participants, intervention or concept, context, outcomes, study design) [46] will be specified to establish eligibility criteria defined according to the following:

- Types of participants: This study considers all human participant groups (eg, children, youth, and adults) at different level of influence (eg, individual, household, or community) who have been implicated by UA.
- Intervention or concept: For the purpose of this review, UA is defined as food growing initiatives that include the production of edible plants and livestock in urban areas. The review will seek studies that assess UA in all its forms when it is used as an intervention consisting to grow food or raise animals for domestic consumption, local sales, or as a leisure activity.
- Outcomes: The targeted outcomes are a set of determinants of health inspired from Dahlgren and Whitehead [42]. For example, food security, income, social relations, and factors that influence mental or physical health (listed in Table 1).
- Context: To be included, studies must have been conducted in urban settings of a high-, middle-, or low-income country according to the World Bank's income-based country classification [41].
- Type of study: Peer reviewed quantitative or qualitative studies demonstrating one or more effects of UA on health or its determinants will be included. Narratives, essays, gray literature and theses will be excluded. Other systematic studies will not be included in the analysis but the list of their references will be examined to identify relevant studies.

#### Search Strategy

The search strategy has been designed with the help of a library specialist and searches will be performed in the following seven electronic bibliographic databases: PubMed, Embase, MEDLINE (Embase), CINAHL Plus with full text, Academic Search Premier (EBSCO host), CAB Abstract (Ovid), and Web of Science. The outlined keywords in Table 1 and their alternative terms will be searched in the index terms, title, and abstract (tiab) of each database. In case a keyword is not found in the index terms, it will be substituted by its alternative term or a synonym in the index search and will be searched in titles and abstracts only. For example, in PubMed, the index is the medical subject heading (MeSH). The word *food security* does not appear as a MeSH, so in the search for MesH, we will use *food supply* as an alternative but the keyword *food security* will also be searched as it is written in the titles and abstracts. Boolean operators *OR* will also be used to combine individual keywords while the Boolean operator *AND* will be used to combine sets of keywords (eg, the words urban agriculture/urban farm or city agriculture/city farm, are searched as following: (urban *OR* city) *AND* (agriculture *OR* farm). An example of the complete search strategy used on PubMed is described in Table 1. This strategy will then be adapted to the other databases using the according syntax and proximity operators.

**Table 1:** Example of search strategy used on PubMed and adapted to other bibliographic databases

Category, number, and keywords			Index terms or search-field descriptors
**Outcome measures**			
	1	Food supply	Mesh
	2	Food security	Tiab
	3	Food insecurity	Tiab
	4	Food access	Tiab
	5	Food availability	Tiab
	6	Food quality	Mesh:NoExp, tiab
	7	Food safety	Mesh:NoExp, tiab
	8	Food contamination	Mesh:NoExp, tiab
	9	Food	Mesh:NoExp
	10	Health* food	Tiab
	11	Income	Mesh:NoExp, tiab
	12	Cost savings	Mesh:NoExp, tiab
	13	Poverty alleviation	Tiab
	14	Nutritional status	Mesh:NoExp, tiab
	15	Nutrient deficiency	Tiab
	16	Fruit and vegetable intake	Tiab
	17	Fruit and vegetable consumption	Tiab
	18	Fruits and vegetables	Tiab
	19	Vegetables	Mesh:NoExp
	20	Fruit	Mesh:NoExp
	21	Fruit? Intake	Tiab
	22	Vegetable? Intake	Tiab
	23	Diet	Mesh:NoExp, tiab
	24	Dietary diversity	Tiab
	25	Malnutrition	Mesh:NoExp, tiab
	26	Undernutrition	Tiab
	27	Overweight	Mesh:NoExp, tiab
	28	Obesity	Mesh:NoExp, tiab
	29	Quality of life	Mesh:NoExp, tiab
	30	Healthy lifestyle	Mesh:NoExp, tiab
	31	Exercise	Mesh:NoExp
	32	Physical activity	Tiab
	33	Leisure activity	Mesh:NoExp
	34	Leisure	Tiab
	35	Well-being	Tiab
	36	Interpersonal relations	Mesh:NoExp, tiab
	37	Social capital	Tiab
	38	Personal development	Tiab
	39	Empowerment	Tiab
	40	Education	Mesh:NoExp
	41	Nutrition education	Tiab
	42	Civic engagement	Tiab
	43	Community engagement	Tiab
	44	Horticultural therapy	Mesh
	45	Therapeutic garden	Tiab
	46	Mental health	Mesh:NoExp, tiab
	47	Dementia	Mesh:NoExp, tiab
	48	Stress psychological	Mesh:NoExp
	49	Stress	Tiab
	50	Perceptions of life	Tiab
	51	Cultural connection	Tiab
	52	Violence	Mesh:NoExp
	53	Depression	Mesh:NoExp
	54	Security perception	Tiab
	55	Health risk	Tiab
	56	Resilience	Tiab
	57	Pain	Mesh:NoExp, tiab
**Intervention/Concept**			
	58	Agriculture	Mesh:NoExp, tiab
	59	Food production	Tiab
	60	Gardening	[Mesh]
	61	Community garden*	Tiab
	62	Farm*	Mesh, tiab
	63	Allotment$	Tiab
	64	Horticultur*	Tiab
	65	Rooftop$	Tiab
	66	Home garden*	Tiab
	67	School garden*	Tiab
**Context**			
	68	Cities	Mesh:NoExp
	69	City	Tiab
	70	Urban	Tiab
	71	Metropol*	Tiab
	72	Suburban	Tiab
	73	Town	Tiab

**Data extraction for analysis (type of data and variables)**

**Reference**

- Author
- Year

**Study location**

- City, country
- Country income level

**Population**

- Type of participants (individual, household, community)
- Characteristics of participants (age; sex; children, youth, adults)

**Type of study**

- Study purpose
- Study design
- Outcomes measured

**Results**

- Type of impacts (beneficial, adverse)
- Results of study

### Selection of Relevant and Reliable Studies

Due to a limited accessibility of UA scientific papers prior the 1980s, the search will be restricted to articles published between 1980 and 2017. Titles in languages other than English, French and Spanish will be excluded in the selection phase. All identified publications will be transferred to EndNote (X8, Thomson Reuters) and articles whose publication dates and languages do not meet our requirements will be removed. All remaining publications will be transferred to an online systematic review software (DistillerSR, Evidence Partners, Ottawa, Canada), to remove duplicates and for title and abstract screening by two independent reviewers. The full text of eligible articles will be screened by two independent reviewers according to the following inclusion criteria:

- Relevance: The study must be relevant to the question and objectives of our research. It will be considered relevant if it demonstrates one or more beneficial or adverse impacts of UA on human health or its determinants.
- Study design: To be included into the scoping review, the study must also present data collected from human participants. Furthermore, the design of the study must be appropriate to answer the studied research questions. Studies that report environmental impacts will be considered only if they report effects on humans (eg, study on soil contamination will not be included unless it reports the effects of soil contamination on human health).

A list of all excluded articles at this stage will be provided with the reasons for exclusion. The reference lists of included studies will also be reviewed to identify relevant studies. The identified studies will be assessed with the same eligibility criteria to validate their inclusion or exclusion. Final inclusion of the publications will be discussed by the two reviewers and any disagreement on the inclusion or exclusion will be resolved by consensus.

#### Study Quality Assessment

The quality of the included studies will be evaluated using the criteria of the Effective Public Health Practice Project (EPHPP) guide for quantitative studies, and the qualitative study evaluation criteria of Wallace et al [47] used by Ohly et al [39] for the assessment of the quality of qualitative studies. The evaluation of the quality of the studies, in both cases, will take into account the risks of bias in the methodologies of the studies. Thus, any evaluated study with a high risk of bias will be reported in the results section.

### Collating, Summarizing and Reporting the Results

Data as described in Textbox 1 will be extracted from the included articles and the results will be presented in a way to identify the main areas of interest and gaps in the literature on UA impacts.

Once this information is extracted, the results will then be presented in two forms to make a narrative account of the literature [43]. As a first step, a numerical analysis will be presented in the form of a diagram [48] that will highlight the measured outcomes—determinants of health according to number, the nature, and the geographical distribution of the included studies. In a second step, the studies will be grouped according to the category and characteristics of studied participants (individuals, households, and communities; age and sex) to make comparisons, identify contradictions in evidence, methodology, and find research gaps.

## Results

The findings are expected to identify research gaps that will inform needs for UA research in specific fields (eg, mental health), among certain population groups (eg, adults) or within different economic contexts (eg, low-, middle- or high-income countries). Furthermore, the findings are expected to identify knowledge gaps and direct future research needs.

## Discussion

To our knowledge, this scoping study is the first of its kind to explore both beneficial and adverse impacts of UA on health determinants. Other systematic studies have already provided valuable information on specific benefits of UA. However, in the current context of urbanization and climate change where health and environmental challenges are related to food production in cities, it is obvious that the adverse impacts of UA are a concern [49]. Therefore, the identification of evidence that only include beneficial impacts of UA, does not allow an objective analysis to draw conclusions on its impacts. With our findings, we hope to bring a set of elements that allow a better understanding when defining the advantages and disadvantages of the UA as an intervention.

This study will highlight the state of research on the association between UA and health. A holistic approach that considers beneficial and adverse effects of UA, may inform better public policies and target intervention populations. The scoping review will allow for a better understanding of the contributions or consequences of UA on specific determinants of health. It may also be used by policy makers to target indicators that can help better evaluate UA as an intervention that directly impacts individuals, households, or communities. Such approach will also serve to inform urban planning decisions where the role of agricultural production has not always been evident [50].

## Abbreviations

MeSH: medical subject headings
PICOS: participants, intervention or concept, context, outcomes, study design
UA: urban agriculture
